# MRI-derived g-ratio and lesion severity in newly diagnosed multiple sclerosis

**DOI:** 10.1093/braincomms/fcab249

**Published:** 2021-11-03

**Authors:** Elizabeth N York, Sarah-Jane Martin, Rozanna Meijboom, Michael J Thrippleton, Mark E Bastin, Edwin Carter, James Overell, Peter Connick, Siddharthan Chandran, Adam D Waldman, David P J Hunt, Future-MS Consortium, Amit Akula, Javier Carod Artal, Sergio Baranzini, Fiona Barret, Mark Bastin, Christine Batchelor, Emily Beswick, Fraser Brown, Siddharthan Chandran, Jessie Chang, Yingdi Chen, Shuna Colville, Peter Connick, Denise Cranley, Rachel Dakin, Baljean Dhillon, Elizabeth Elliot, James Finlayson, Peter Foley, Stella Glasmacher, Angus Grossart, Haane Haagenrud, Katarzyna Hafezi, Emily Harrison, Adil Harroud, Sara Hathorn, Tracey Hopkins, David Hunt, Aidan Hutchinson, Kiran Jayprakash, Matt Justin, Agniete Kampaite, Patrick Kearns, Gwen Kennedy, Michaela Kleynhans, Julian Ng Kee Kwong, Juan Larraz, Kathryn Love, Dawn Lyle, James MacDonald, Niall MacDougall, Lesley Macfarlane, Beverly Maclennan, Alan Maclean, Margaret Ann MacLeod, Nicola Macleod, Don Mahad, Sarah Jane Martin, Lynn McMahon, Ian Megson, Rozanna Meijboom, Daisy Mollison, Mary Monaghan, Lee Murphy, Katy Murray, Judith Newton, Jonathan O’Riordan, David Perry, Suzanne Quigley, Adam Scotson, Amy Stenson, Michael Thrippleton, Maria Valdez Hernandez, Adam Waldman, Christine Weaver, Stewart Webb, Belinda Weller, Anna Williams, Stewart Wiseman, Charis Wong, Michael Wong, Elizabeth York

**Affiliations:** 1 Centre for Clinical Brain Sciences, University of Edinburgh, Edinburgh EH16 4SB, UK; 2 Department of Neurosciences, University of Glasgow, Glasgow G51 4LB, UK; 3 Institute of Genetics and Molecular Medicine, University of Edinburgh, Edinburgh EH4 2XU, UK; 4 UK Dementia Research Institute, University of Edinburgh, Edinburgh EH16 4SB, UK; 5 Anne Rowling Clinic, University of Edinburgh, Edinburgh EH16 4SB, UK

**Keywords:** multiple sclerosis, MRI, neurofilament, myelin, g-ratio

## Abstract

Myelin loss is associated with axonal damage in established multiple sclerosis. This relationship is challenging to study *in vivo* in early disease. Here, we ask whether myelin loss is associated with axonal damage at diagnosis by combining non-invasive neuroimaging and blood biomarkers. We performed quantitative microstructural MRI and single-molecule ELISA plasma neurofilament measurement in 73 patients with newly diagnosed, immunotherapy naïve relapsing–remitting multiple sclerosis. Myelin integrity was evaluated using aggregate g-ratios, derived from magnetization transfer saturation and neurite orientation dispersion and density imaging diffusion data. We found significantly higher g-ratios within cerebral white matter lesions (suggesting myelin loss) compared with normal-appearing white matter (0.61 versus 0.57, difference 0.036, 95% CI: 0.029–0.043, *P* < 0.001). Lesion volume (Spearman’s rho r_s_= 0.38, *P* < 0.001) and g-ratio (r_s_= 0.24, *P* < 0.05) correlated independently with plasma neurofilament. In patients with substantial lesion load (*n* = 38), those with higher g-ratio (defined as greater than median) were more likely to have abnormally elevated plasma neurofilament than those with normal g-ratio (defined as less than median) [11/23 (48%) versus 2/15 (13%), *P* < 0.05]. These data suggest that, even at multiple sclerosis diagnosis, reduced myelin integrity is associated with axonal damage. MRI-derived g-ratio may provide useful additional information regarding lesion severity and help to identify individuals with a high degree of axonal damage at disease onset.

## Teaser

York et al. simultaneously measured g-ratio and plasma neurofilament in 73 relapsing–remitting multiple sclerosis patients at diagnosis using advanced MRI and single-molecule ELISA. They demonstrate that g-ratio of cerebral white matter lesions varies at diagnosis, and show that high g-ratio of lesions is associated with elevated plasma neurofilament.

## Graphical Abstract

**Graphical Abstract:**
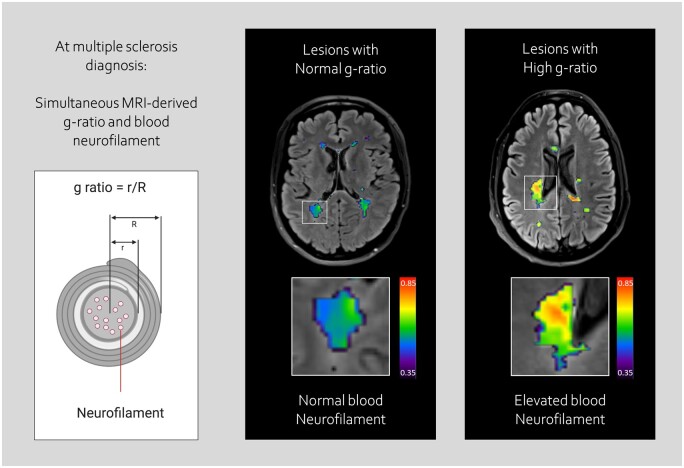

## Introduction

Multiple sclerosis is an unpredictable neurological disease characterized by immune-mediated myelin loss and axonal degeneration.^1^ Neuropathological studies of multiple sclerosis lesions^2^ have demonstrated a link between loss of myelin and axonal transection, and the latter is considered an important substrate of long-term disability.^2^^,^^3^ Optimizing earlier diagnosis and treatment opportunities requires improved understanding of the influence of demyelination on neurodegeneration at the earliest potential point of therapeutic intervention.

**Figure 1:**
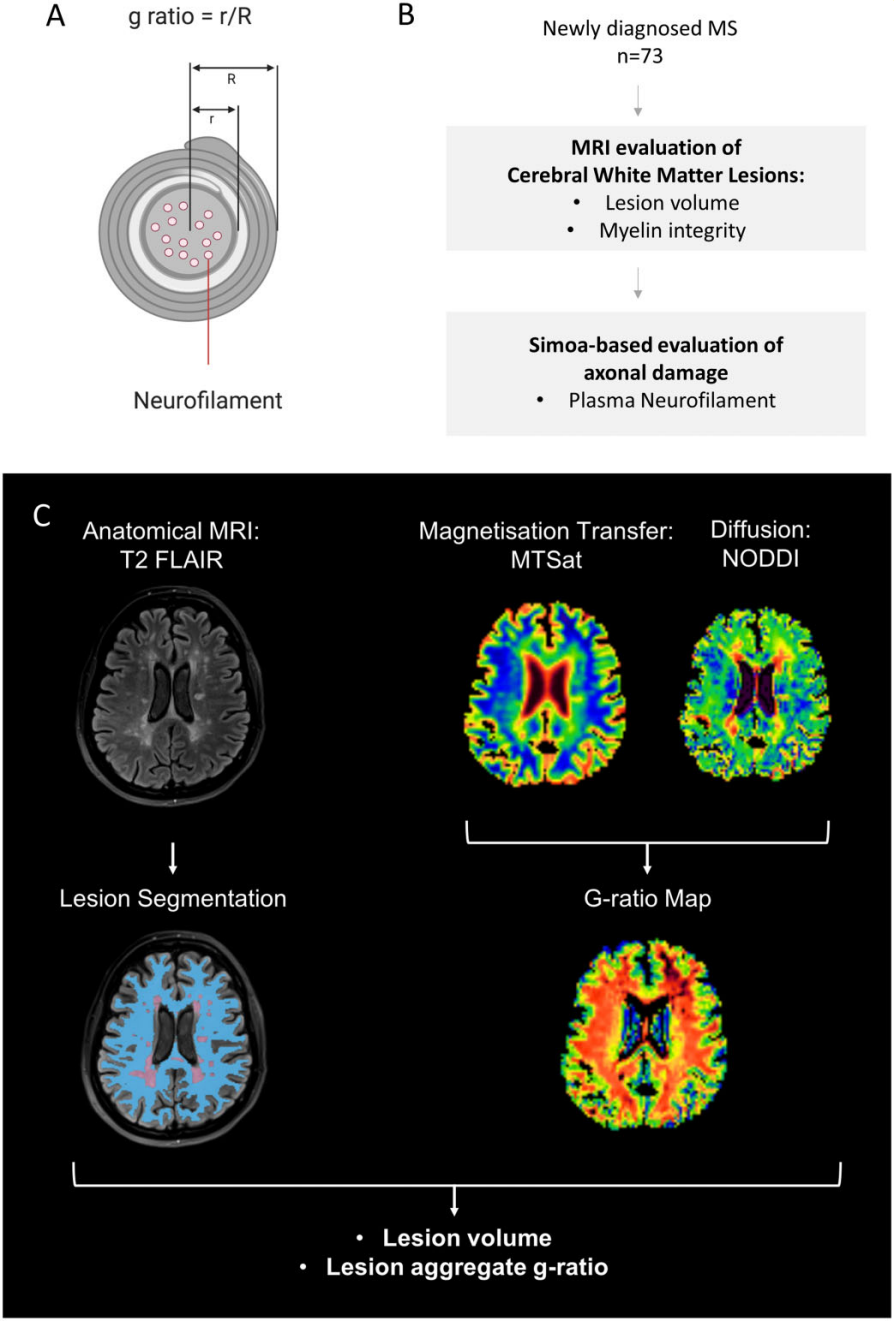
**Study overview: determining g-ratio of cerebral white matter lesions at multiple sclerosis diagnosis**. Schematic cross-section of a myelinated axon, shown in **A**, showing definition of g-ratio, and location of neurofilaments. The g-ratio is defined as the ratio of the inner axonal radius to the outer fibre radius with the myelin sheath. Neurofilaments are critical structural proteins within the axon that are released into CSF and plasma in the context of axonal damage. Simultaneous evaluation of myelin integrity and axonal damage, shown in **B**, was performed in a cohort of 73 patients with newly diagnosed relapsing–remitting multiple sclerosis. A multimodal brain MRI protocol, shown in **C**, was acquired on a 3T clinical system (Prisma, Siemens, Erlangen.de), which included magnetization transfer saturation (MTsat) and multishell water diffusion MR acquisitions. WML were defined as hyperintensities on T_2_ Fluid-Attenuated Inversion Recovery and NAWM was segmented as described in the text. In this example, NAWM is shown in blue and WML in purple. Myelin integrity can be quantified as an ‘aggregate’ MR g-ratio, derived from MTsat and diffusion-derived neurite orientation dispersion and density imaging (NODDI) data, according to the equations shown in the text and Supplementary methods. The segmentation and g-ratio approaches were combined to determine aggregate g-ratios from both WML and NAWM.

Advances in MR imaging and ultrasensitive protein detection technologies offer new opportunities for quantified *in vivo* study of myelin integrity and axonal damage at critical timepoints in multiple sclerosis, such as the point of diagnosis. For example, quantitative magnetization transfer (MT) and water molecule diffusion-derived MRI methods allow non-invasive evaluation of microstructural integrity and myelin pathology,^4–6^ and have the potential to provide insights into myelin loss in multiple sclerosis *in vivo.* The g-ratio is defined as the ratio of the inner to outer radius of the myelin sheath (Fig. 1A) and provides a specific measure of myelin integrity.^7^ An MR-derived ‘aggregate g-ratio’ can be estimated in defined regions of the brain by combining MT and diffusion biomarker data.^8^^,^^9^ The MR g-ratio has previously been validated against tissue measures in both healthy controls and people with multiple sclerosis. Abnormally high g-ratios indicate disruption of myelin.^10^^,^^11^

Ultrasensitive digital ELISA technologies such as single-molecule arrays allow accurate quantification of proteins associated with axonal damage in blood at subfemtomolar concentrations.^12^ Neurofilaments, a key component of the neuronal cytoskeleton (Fig. 1A), are released during axonal damage; abnormally elevated levels within CSF and blood are associated with axonal damage and poorer long-term multiple sclerosis neurological outcomes.^13^ Ultrasensitive neurofilament quantification using single molecule array can be simultaneously combined with advanced neuroimaging, to provide biological insights into mechanisms of axonal loss in multiple sclerosis.

We therefore combined imaging and blood biomarkers to examine the relationship between myelin integrity and axonal damage in patients with newly-diagnosed relapsing–remitting multiple sclerosis (RRMS), prior to initiation of disease-modifying therapies (DMTs).

## Materials and methods

### Participants

We performed paired MRI and blood sampling in 73 patients (Fig. 1B), recruited as part of the Future-MS study (https://future-ms.org/). All provided written consent, were recruited within 6 months of diagnosis of RRMS and were treatment naïve. Overarching study design and ethical approval for the Future-MS study were sought and obtained by the Future-MS study team at the Anne Rowling Regenerative Neurology Centre (ethical approval reference REC 15/SS/023).

### MR imaging data acquisition

Imaging was performed on a 3.0-T MR system (Prisma; Siemens Healthcare, Erlangen, Germany) with a 32-channel head coil. Technical details of data acquisition and preprocessing are found in the Supplementary material and Supplementary Table 1.

### Derivation of g-ratio using MRI

An overview of g-ratio mapping within white matter lesions (WML) and normal-appearing white matter (NAWM) is shown in Fig. 1C. Hyperintense WML on T_2_ Fluid-Attenuated Inversion Recovery were automatically segmented using a combination of FSL (6.0.1, http://www.fmrib.ox.ac.uk/fsl) and an intensity-based thresholding method^14^; following automated segmentation, all lesion masks were reviewed and manually edited by a trained technician under supervision of a post-doctoral imaging scientist (5 years experience; R.M.) and an experienced neuroradiologist (20 years experience; A.D.W.).

G-ratio was derived according to the equation^15^:

$$g=\sqrt{{\left(1+\frac{\mathrm{MVF}}{\mathrm{AVF}}\right)}^{-1}}$$

where MVF is the myelin volume fraction and AVF is the axonal volume fraction. MVF was calculated from magnetization transfer saturation (MTsat) imaging and AVF was calculated from neurite orientation dispersion and density imaging described below. Parametric g-ratio maps were masked using NAWM and WML segmentations to give regional mean g-ratio values (further details in Supplementary methods).

### Calculation of MVF and AVF

The MVF was calculated from MTsat,^16^ assuming a linear relationship,^9^ as:

$$\mathrm{MVF}=\delta_{\mathrm{app}}*k$$

where *k* is a constant calibrated from healthy control data (see Supplementary methods).

The AVF was calculated from neurite orientation dispersion and density imaging data^17^ and scaled according to the MVF as^15^:

$$\mathrm{AVF}=(1-\mathrm{MVF})(1-v_{\mathrm{iso}})v_{\mathrm{ic}}$$

where $v_{\mathrm{iso}}$ and $v_{\mathrm{ic}}$ are the isotropic and restricted signal fractions derived from the neurite orientation dispersion and density imaging model.

### Quantification of plasma neurofilament light-chain using single-molecule ELISA

Plasma neurofilament levels were measured using a NF-light assay (4-PLEX ‘A’ Kits Quanterix) and a single molecule array SR-X^TM^ instrument in 73 patients and 63 healthy age- and sex-matched controls. Samples were analysed in duplicate, with researchers blinded to sample identification. All results were above the lower limit of quantification, with coefficient of variation < 20%. Normal ranges were derived from the matched healthy controls. An ‘elevated’ neurofilament light-chain (NFL) result was considered a plasma NFL value more than 3 SD higher than the mean of the healthy control population.

### Statistical analyses

Statistical analyses were performed using R (RStudio version 1.2.5033) and GraphPad Prism (version 8.4.1). Histogram analysis and a Wilcoxon signed-rank test was used to assess the difference between aggregate g-ratio values from WML and NAWM (α = 0.05). The difference in NfL levels between multiple sclerosis patients and healthy controls was measured with a Mann–Whitney U test (α = 0.05). Spearman’s rho was used to test for an association between g-ratios and NfL levels and, to examine the influence of lesion load, between intracranial volume-corrected lesion volume and NfL levels (α = 0.05). Follow-up tests were performed with Fisher’s exact test of independence. In addition to these tests for association, and bearing in mind previous studies suggesting a lesion volume threshold for detecting axonal damage,^12^ we stratified patients by lesion load to determine the influence of g-ratio on a patient’s risk of having abnormally high plasma neurofilament levels. The following definitions were used: Low lesion load (≤0.5% intracranial volume -corrected Whole Brain Lesion Volume) versus Substantial lesion load (>0.5%) lesion load, and Normal WML g-ratio (≤ median) versus Abnormal WML g-ratio (>median). The threshold value of <0.5% intracranial volume -corrected Whole Brain Lesion Volume was chosen as it was closest to the median lesion volume. The same analyses were also repeated with lesion volume thresholds of <0.4% and <0.6%. Subgroup differences were assessed with Fisher’s exact tests (α = 0.05 for all analyses).

### Data availability statement

The data that support the findings of this study are available from the corresponding author, upon reasonable request.

## Results

Seventy-three individuals with RRMS were included in the study. The cohort was reflective of a newly diagnosed RRMS cohort, and was predominantly female, with a median age of 33 years (interquartile range 28–45 years) and a median EDSS score of 2 (range 0–6). All patients were included within 6 months of diagnosis and whilst treatment naïve (see Table 1 and Supplementary Fig. 1).

**Table 1:** Baseline demographics and disease severity of newly diagnosed RRMS patient recruited to the study

	Future-MS advanced myelin imaging study
Participants	73
Sex ratio F: M	3.6
Median age at diagnosis (range)	33 (28–45)
Median EDSS (range)	2 (1.5–3)

**Figure 2:**
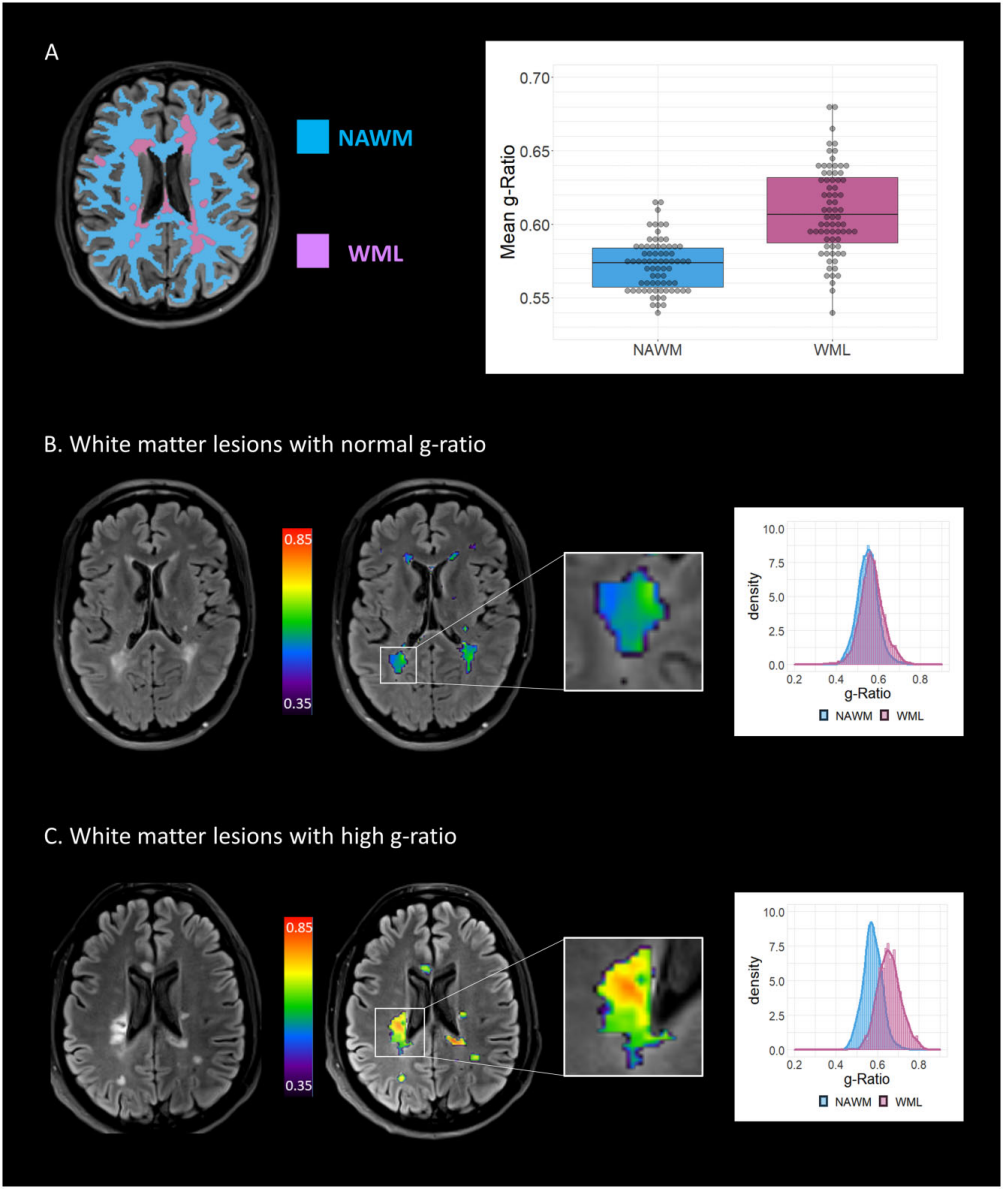
**G-ratio in cerebral white matter lesions at MS diagnosis**. **A** shows segmentation strategy for anatomical MRI scans, dividing white matter into white matter lesions (WML, purple) and normal-appearing white matter (NAWM, blue). The g-ratio is increased in lesions compared to NAWM in relapsing–remitting multiple sclerosis patients (0.61 versus 0.57, difference: 0.036, 95% CI: 0.029–0.043, *P*<0.001 paired t-test). **B** shows representative patient with normal g-ratio within WML. On the left, the T_2_ Fluid-Attenuated Inversion Recovery image is shown, with hyperintense WML consistent with multiple sclerosis. The aggregate g-ratio from these lesion segmentations was evaluated (right-hand MRI, g-ratio denoted by colour scale), leading to a histogram where the distribution of g-ratio values between NAWM (blue) and WML (purple) could not be distinguished. **C** shows representative patient with high g-ratio within WML. The lesion shown has a high g-ratio (right-hand MRI) and the resulting individual histogram from the patient shows separation of g-ratio distributions derived from NAWM (blue) and WML (purple).

We first looked at the difference in g-ratio between NAWM and WML in newly diagnosed RRMS patients. G-ratios within WML (median 0.61) were significantly higher than in NAWM (median 0.57, *P* < 0.001, Z-value 0.80, Fig. 2A). WML g-ratio varied widely (range: 0.54–0.68, Fig. 2A), and identified individuals with both normal and elevated WML g-ratio at diagnosis (Fig. 2B and C), suggesting the potential for g-ratios to identify a subset of patients with severe myelin disruption at diagnosis. An example of two patients with similar lesion volume determined by anatomical MRI, but distinctive aggregate g-ratios, is shown in Fig. 2B. The first individual has similar g-ratios in both WML and NAWM, suggesting preserved myelin integrity within lesions (Fig. 2B). The second individual has higher aggregate g-ratio in WML than NAWM, suggesting more severe myelin disruption within lesions (Fig. 2C).

**Figure 3:**
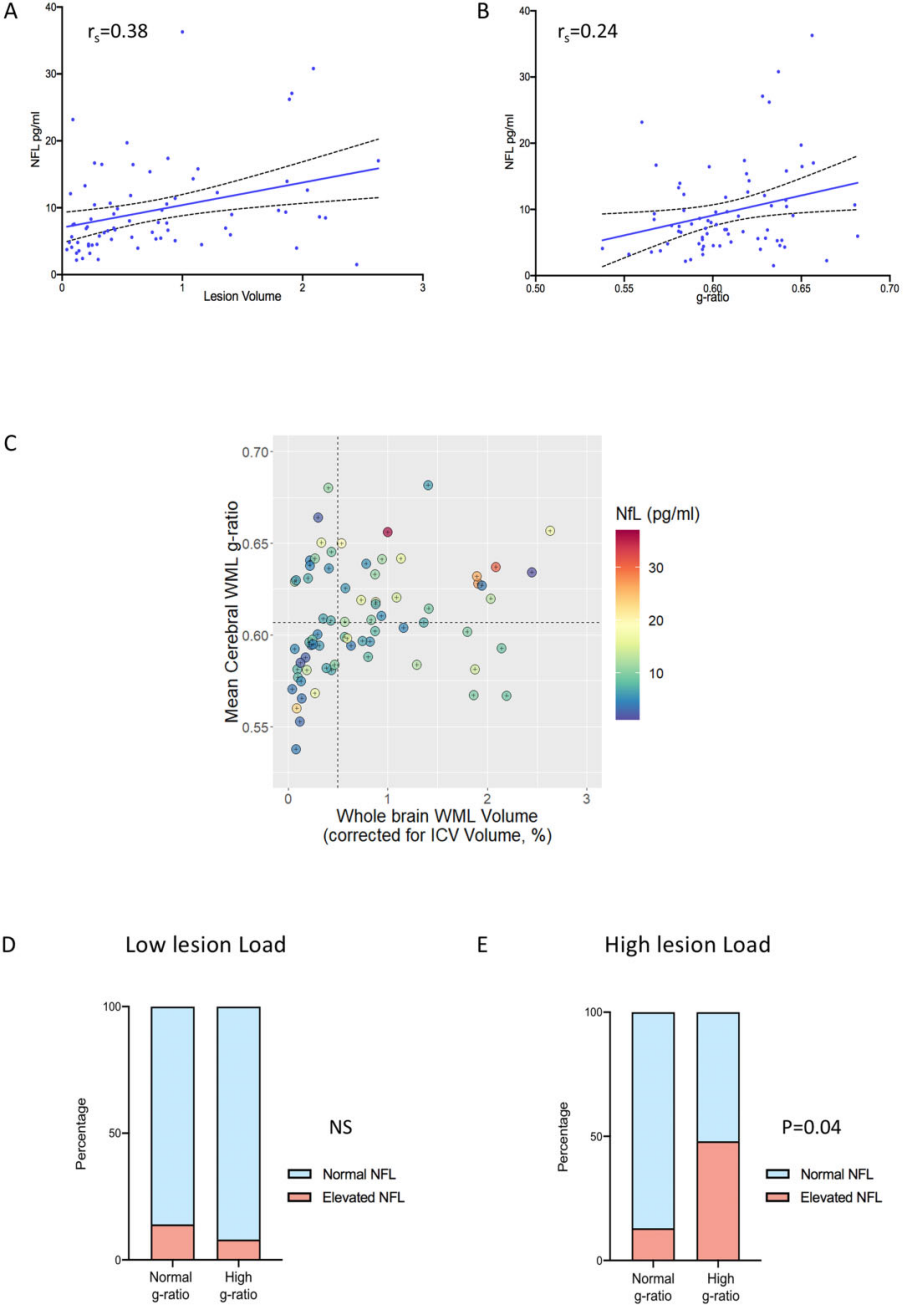
**Influence of cerebral white matter lesion g-ratio on plasma neurofilament levels at multiple sclerosis diagnosis**. **A** shows plasma neurofilament levels are associated with intracranial volume (ICV)-corrected WML volume, Spearman’s rho = 0.38, *P* = 0.001. **B** shows plasma neurofilament levels are associated with WML g-ratio, Spearman’s rho = 0.24, *P* = 0.05. **C** shows relationship between whole brain WML volume (as a percentage of ICV), lesional g-ratio and plasma neurofilament in study subjects. Dotted lines represent cut-offs used to define categories (low lesion load versus high lesion load, and normal g-ratio versus high g-ratio). In patients with low WML volume at diagnosis, there was no significant difference between the proportion of patients with abnormally raised plasma neurofilament [3/22 versus 1/13, no significant difference in proportion (N.S.), Fisher’s Exact test], shown in **D**. In patients with substantial WML volume at diagnosis, a higher proportion of patients (48%) with a high lesional g-ratio had abnormally raised neurofilament compared to those with normal g-ratios (11/23 versus 2/15, *P* < 0.05, Fisher’s Exact test), shown in **E**.

We then asked whether these MRI-determined measures of myelin disruption within WMLs were associated with increased plasma neurofilament concentration. Single molecule array analysis showed significantly higher plasma neurofilament concentrations in newly diagnosed multiple sclerosis patients compared with age- and sex-matched control subjects (7.6 versus 4.5 pg/ml, 95% CI: 2.7–6.3, *P* < 0.001, Supplementary Fig. 2). Seventeen out of 73 patients (23%) had plasma neurofilament levels that were elevated compared to the matched healthy control population. Plasma neurofilament concentration correlated with WML volume (r_s_ = 0.38, *P* < 0.001, Fig. 3A). A significant, though weaker, association was observed with g-ratio within WML (r_s_ = 0.24, *P* < 0.05, Fig. 3B). In order to establish the specific value of g-ratio measurements as a marker of myelin loss, we repeated this analysis with other microstructural MRI markers, and observed no significant association between neurofilament and magnetization transfer ratio, MTsat or T1app, which provides an approximation of T_1_ recovery time (Supplementary Fig. 5). We did not identify any association between MR measures and disability scores at baseline or Year 1 (Supplementary Table 2).

We stratified patients according to lesion burden at diagnosis (Fig. 3C). In patients with low WML load at diagnosis (*n* = 35), the proportion of patients with elevated plasma NFL was the same in the normal g-ratio and high g-ratio groups [3/22 (14%) versus 1/13 (8%), N.S., Fig. 3D]. However, in patients with a substantial WML load at diagnosis (*n* = 38), individuals with an elevated WML g-ratio were more likely to have raised plasma neurofilament compared with individuals with normal g-ratio [11/23 (48%) versus 2/15 (13%), *P* < 0.05, Fig. 3E]. There were no significant differences in WML volume or anatomical location of lesions between the high and normal g-ratio groups (Supplementary Figs 3 and 4). This effect survived if lower (0.4%) or higher (0.6%) thresholds for WML volume were selected (Supplementary Table 3). Therefore, in RRMS patients with otherwise indistinguishable anatomical MRI features, determining g-ratios within WMLs can identify patients with more severe lesions that are associated with higher levels of axonal damage.

## Discussion

This is the first study to combine non-invasive microstructural MR imaging and blood biomarkers to evaluate myelin and axonal integrity at the point of RRMS diagnosis. Our cohort is reflective of ‘typical’ RRMS patients at the point of diagnosis and before the initiation of disease-modifying treatments.

We found that aggregate MRI g-ratios were higher in cerebral multiple sclerosis lesions compared with surrounding NAWM, in keeping with comparative loss of myelin integrity within lesions. We also, however, observed wide variation in lesional g-ratio within our clinically homogeneous cohort, indicating heterogeneity in the degree of myelin disruption within lesions that are otherwise indistinguishable on conventional MRI. Determining g-ratios in multiple sclerosis lesions therefore adds specificity to MRI, and may provide biological and clinical insight into differing severity of lesions that appear similar on conventional imaging.

Quantification of axonal damage using elevated plasma/serum neurofilament is clinically important because of a strong association with risk of future disability.^12^^,^^13^ In this cohort, neurofilament levels are elevated at the point of diagnosis of RRMS compared with healthy controls, in line with previous evidence that axonal damage occurs early in the disease course.^13^ Our finding of a positive association between neurofilament levels and WML volume (derived from anatomical MRI data) further confirms the recently observed association between MRI-visible lesion burden and plasma neurofilament as a marker of axonal degeneration.^12^

Furthermore, our data suggest that individuals with high WML g-ratios have greater levels of axonal damage reflected in elevated plasma neurofilament levels. MRI g-ratios are derived by combining AVF measures from neurite orientation dispersion and density imaging and MVF from MTsat to provide an estimate of myelin thickness. A number of previous studies have identified an association between T_1_ and magnetization transfer ratio values,^18^ and myelin loss, including paired MRI–neuropathology studies.^18^^,^^19^ Our analysis, however, found significant correlation between lesional g-ratio and elevated serum neurofilament, whereas MT and T_1_ measures alone showed no such association with serum neurofilament. The degree to which g-ratio may provide a more specific surrogate measure of demyelination in early RRMS, and how individual MT and diffusion measures reflect varying proportions of myelin and axonal damage, warrants further evaluation.

We further explored this association between surrogate markers of demyelination and axonal damage, based on the previous neuropathological demonstration of a strong association between demyelination and axonal loss in lesions in advanced multiple sclerosis.^2^^,^^20^ Although linear regression would be one method to explore the relationship between MRI markers and serum biomarkers, our data did not meet the assumptions behind this approach. A threshold effect had been previously noted in terms of neurofilament detection where there is a low volume of damaged myelin,^12^ and this is particularly relevant to our study, given that lesion load at the point of diagnosis is often very low, and patients with highly active disease (who need urgent DMT initiation) are excluded from the study.

We therefore hypothesized that the relationship between g-ratio and neurofilament would be stronger in patients with substantial lesion load, and examined correlation using a nonparametric approach with dichotomized datasets using thresholds around the median. This analysis demonstrated that increased g-ratio within WML is associated with increased plasma neurofilament in patients with substantial lesion load. The absence of detectable association where WML load is low also supports this hypothesis. The choice of WML threshold for dichotomizing the groups has the potential for bias; however, a sensitivity analysis suggests the association between g-ratio and plasma neurofilament in subjects with substantial lesion load is not significantly influenced by this threshold value.

MRI-derived g-ratio may therefore provide non-invasive information on both myelin integrity and, indirectly, axonal damage, additional to conventional MRI measures. Our results accord with classic neuropathological studies, which show that loss of myelin integrity within lesions is associated with axonal transection in established/late-stage multiple sclerosis,^2^ and further suggest that this relationship may be established at the time of RRMS diagnosis.

We did not detect any association between g-ratio or neurofilament and clinical disability scores. This is unsurprising as such associations typically emerge over much longer intervals than the single year covered in this analysis. Our study will follow these patients up over the long-term and it may be that such clinically relevant disease associations emerge with time.

This study cross-validates imaging and blood biomarkers targeted to specific pathophysiological features, which provide potential surrogates for invasive tissue examination, as used in other disease areas such as neuro-oncology.^21^ This approach is particularly important at diagnosis, which is a critical time for decision-making in RRMS. There is a pressing need to identify patients with a high degree of lesion-associated axonal damage at onset, who may benefit most from highly effective therapeutic interventions.^22^^,^^23^ Early non-invasive detection of myelin destruction within WML may help identify such patients. Such combined biomarker studies also allow pathophysiological models of disease; in this case, the relationship between demyelination and axonal damage, to be tested *in vivo*. A particular advantage of our study is that we have performed paired imaging and serum analyses prior to initiation of DMTs, which are known to influence such biomarkers.

We acknowledge a number of limitations of our work. MRI g-ratios are a derived, model-dependent measure, and may vary with technical factors, including brain location.^9^ In addition, any measurements made relative to NAWM in multiple sclerosis patients may be confounded by microstructural abnormalities in NAWM.^24^ Our cerebral neuroimaging does not capture neurodegeneration in the posterior fossa or spinal cord, which may contribute to neurofilament levels. The MRI protocol also did not include gadolinium contrast-enhanced imaging, and we therefore cannot examine influence of active plaques on transient elevation of neurofilament. Finally, although this represents a substantial multiple sclerosis g-ratio biomarker study, larger comprehensive cohorts with multi-institutional biomarker validation will be needed to identify factors that drive axonal damage in early multiple sclerosis, where lesion load and neurofilament levels are relatively low.^25^

## Conclusions

Loss of myelin integrity, reflected in elevated g-ratio within brain WML, is detected in individuals with RRMS at diagnosis—and is associated with increased axonal damage. The combination of MR-derived g-ratio and blood biomarker of neurodegeneration has the potential to provide both clinically useful information about severity of myelin damage in lesions and early neurodegeneration, with relevance for future therapeutic stratification.

## Supplementary material

Supplementary material is available at *Brain Communications* online.

## Supplementary Material

fcab249_Supplementary_Data

## Appendix I Members of the Future-MS Consortium

The Future-MS consortium includes clinicians, data processors or other Future-MS team members that have contributed to acquisition of data, access to patients or whom have been relied upon for their input. Titles and affiliations are outlined in an appendix to the supplementary files.

Amit Akula, Javier Carod Artal, Sergio Baranzini, Fiona Barret, Mark Bastin, Christine Batchelor, Emily Beswick, Fraser Brown, Siddharthan Chandran, Jessie Chang, Yingdi Chen, Shuna Colville, Peter Connick, Denise Cranley, Rachel Dakin, Baljean Dhillon, Elizabeth Elliot, James Finlayson, Peter Foley, Stella Glasmacher, Angus Grossart, Haane Haagenrud, Katarzyna Hafezi, Emily Harrison, Adil Harroud, Sara Hathorn, Tracey Hopkins, David Hunt, Aidan Hutchinson, Kiran Jayprakash, Matt Justin, Agniete Kampaite, Patrick Kearns, Gwen Kennedy, Michaela Kleynhans, Julian Ng Kee Kwong, Juan Larraz, Kathryn Love, Dawn Lyle, James MacDonald, Niall MacDougall, Lesley Macfarlane, Beverly Maclennan, Alan Maclean, Margaret Ann MacLeod, Nicola Macleod, Don Mahad, Sarah Jane Martin, Lynn McMahon, Ian Megson, Rozanna Meijboom, Daisy Mollison, Mary Monaghan, Lee Murphy, Katy Murray, Judith Newton, Jonathan O’Riordan, David Perry, Suzanne Quigley, Adam Scotson, Amy Stenson, Michael Thrippleton, Maria Valdez Hernandez, Adam Waldman, Christine Weaver, Stewart Webb, Belinda Weller, Anna Williams, Stewart Wiseman, Charis Wong, Michael Wong, Elizabeth York.

## Abbreviations

AVF =: axonal volume fraction
DMT =: disease-modifying therapy
NFL =: neurofilament light-chain
NAWM =: normal-appearing white matter
MTsat =: magnetization transfer saturation
MVF =: myelin volume fraction
RRMS =: relapsing–remitting multiple sclerosis
WML =: white matter lesions
