# Supplementary material for: MRI-derived g-ratio and lesion severity in newly diagnosed multiple sclerosis
Source: Brain Commun. 2021 Nov 3;3(4):fcab249. doi: 10.1093/braincomms/fcab249 (PMC8643503; doi:10.1093/braincomms/fcab249)
Supplement: fcab249_Supplementary_Data [file fcab249_supplementary_data.pdf]

Supplementary Information:

MRI-derived g-ratio and lesion severity in newly diagnosed multiple sclerosis

Contents

Supplementary Methods

Supplementary Figures 1-5

Supplementary Table 1-3

Supplementary Methods

MR Imaging Data Acquisition

MRI Acquisition Parameters are outlined in supplementary table 1.

MR Preprocessing

Hyperintense white matter lesions (WML) on T2 FLAIR (Fluid Attenuated Inversion Recovery) were automatically segmented using a combination of FSL (5.0.1, http://www.fmrib.ox.ac.uk/fsl) and an in-house thresholding method (Figure 1C). Intracranial volumes (ICV) were segmented with FSL and cerebral normal-appearing white matter (NAWM) segmentation masks were obtained with FreeSurfer (6.0, https://surfer.nmr.mgh.harvard.edu/) from the 3D T1-weighted MPRAGE image. Along with the 3D T1-weighted MPRAGE, masks were registered to DW space with FSL FLIRT epi_reg, binarised and NAWM masks were eroded by one voxel to reduce partial volume effects. Segmentations were manually checked and edited where necessary (Figure 2A). The cerebellum was not included due to technical inaccuracies.

MTsat (δ_{app}) and T1app parametric maps were calculated according to Helms et al[1], with in-house code in MATLAB (R2018b, see https://doi.org/10.7488/ds/2965), and registered to the first volume (b0) of the diffusion data with the FSL FLIRT epi_reg script. The magnetisation transfer ratio (MTR) was calculated as:

$$MTR = 100 \cdot \frac{(S_{PD} - S_{MT})}{S_{PD}}$$

The diffusion data were processed with FSL (6.0.1) to extract the brain from the skull, remove bulk motion and eddy-current induced distortions, and register all volumes to the first diffusion volume. The NODDI toolbox (v1.0, mig.cs.ucl.ac.uk, MATLAB R2016b) was used to determine signal fractions from the diffusion MR-visible compartments.

Myelin Volume Fraction: Calculation of k from healthy control data

The constant, *k*, was derived by assuming a g-ratio of 0.7 in the splenium of the corpus callosum from two healthy controls. Healthy controls were scanned twice, one week apart. Data processing followed the same steps as patient data (without WML masks). The splenium mask was derived with FreeSurfer (https://surfer.nmr.mgh.harvard.edu/). The calibration factor was calculated as:

$$k = \frac{1}{\delta_{app}} * (1 - (\frac{1}{(1 + ((\frac{1}{0.7^2}) - 1)(1 - v_{iso})v_{ic})}))$$

The resulting mean k value across both sessions and subjects was 13.921 (Subject 1: 12.1717 and 14.24 ; Subject 2: 15.237 and 13.488, at time-point 1 and 2, respectively).

G-ratio Flat Lesion Maps

In order to assess the impact of lesion location on g-ratio, we created a 2D g-ratio distribution "flat" lesion maps for visual assessment. G-ratio parametric maps were masked with white matter lesion segmentations (FSLmaths), and imported into MATLAB (R2018b) with SPM12 functions. G-ratio values were averaged along the z-axis, creating a 2D array, which roughly approximates an individual's cerebral lesion distribution in the superior-to-inferior axis. Although, anatomically, this distribution will vary to some degree depending on the subject's position in the MR scanner, it allows quick visual assessment of any anterior-posterior bias in the distribution of g-ratio values. A colour-map was overlaid onto the 2D maps and the distribution of lesions and z-direction-averaged g-ratios across the four groups (high vs low lesion load/high vs. normal g-ratio) were compared visually.

A

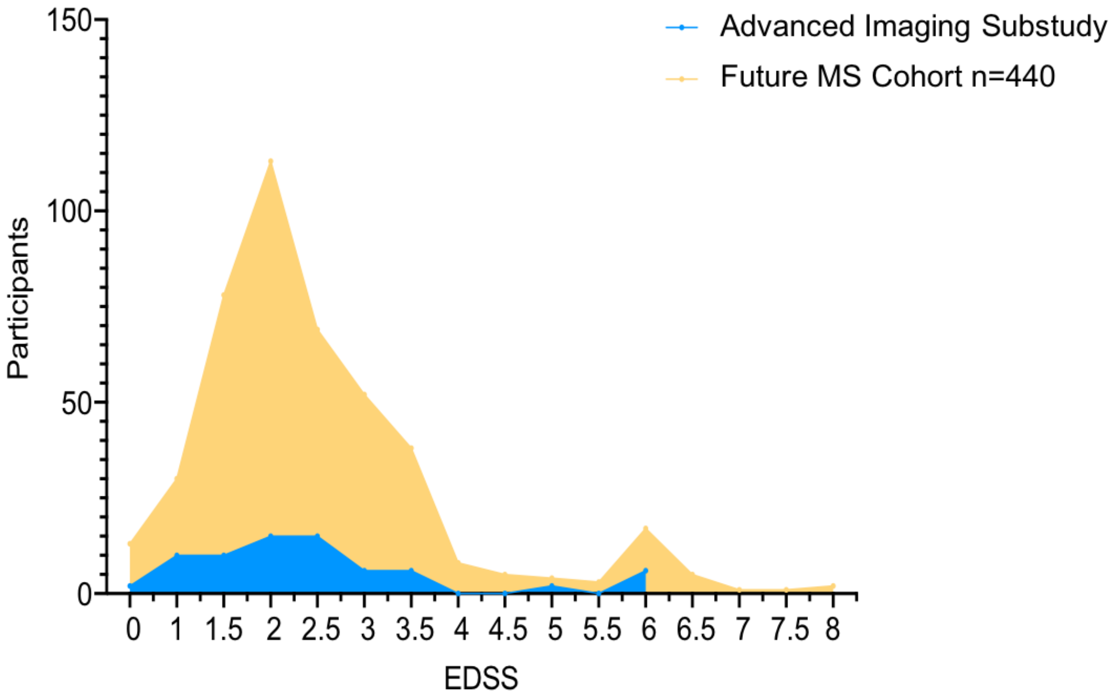

B

	This cohort	Future MS
Participants	73	440
Sex ratio, F:M	3.6	2.82
Age at diagnosis	33 (28-45)	37 (30-45)
Time from symptom onset to study inclusion	34 (12-69)<br>N=67/73	22 (10-62)<br>N=422/440
EDSS at baseline	2 (1.5-3)	2 (1.5-3)

Supplementary Figure 1. Baseline disability and demographics of Advanced Imaging Substudy of the Future MS cohort

EDSS distribution and demographic details of the Future-MS extended imaging substudy (n=73) compared to the Future MS (FMS) study (n=440, which include the 73 imaging substudy participants)

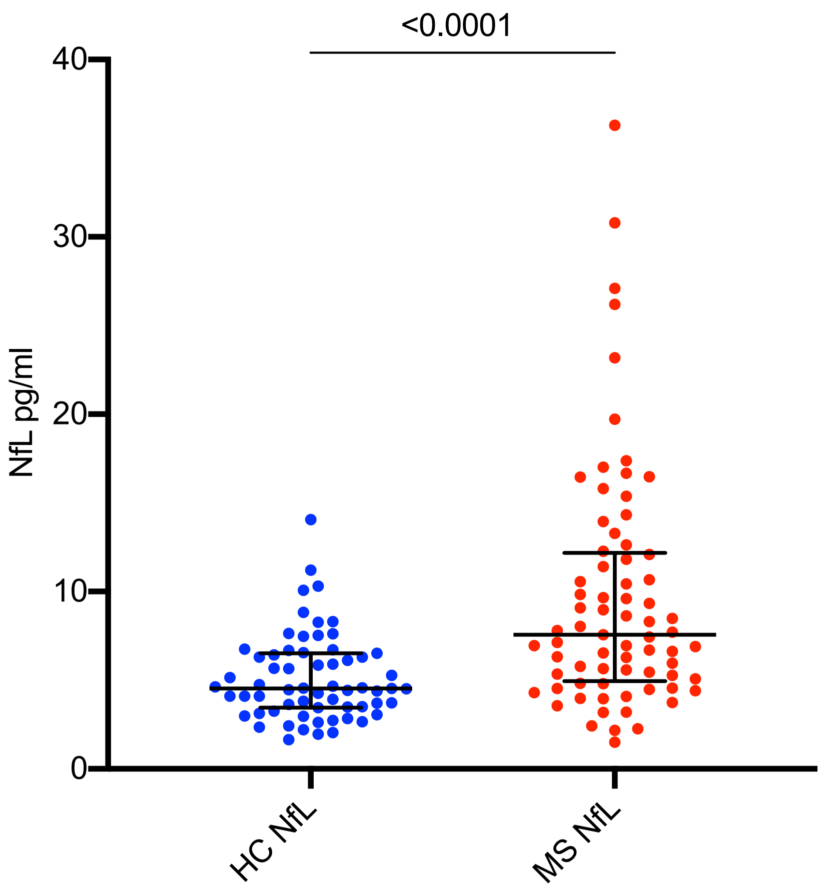

Supplementary Figure 2. Plasma neurofilament levels are elevated in newly diagnosed MS patients compared to age and sex-matched healthy controls (HC) ($p<0.001$, Mann-Whitney U-test)

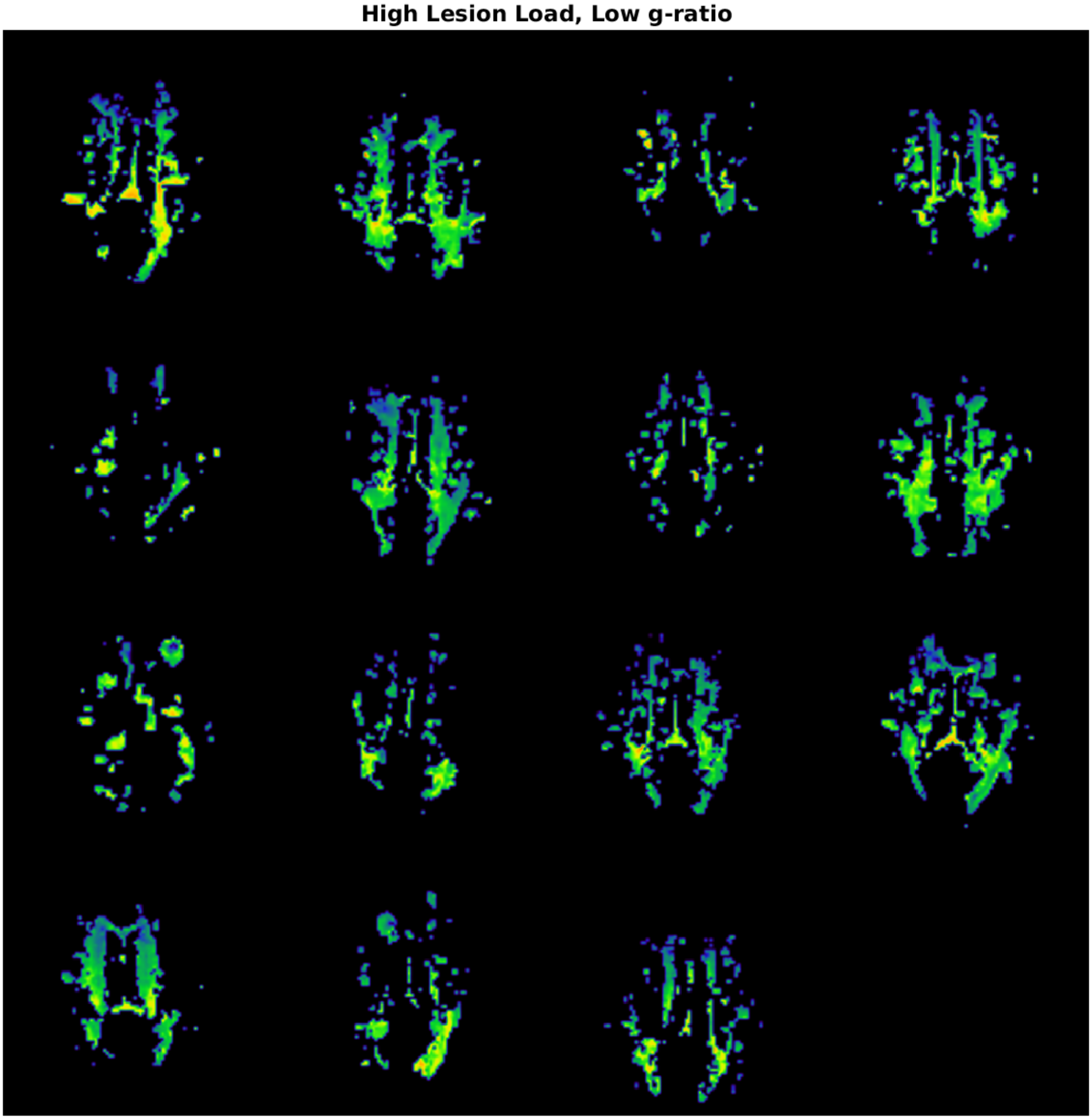

Supplementary Figure 3. Flat map images of individual study participants with high lesion load (Corrected WML volume> 0.5%) and low g-ratio

In order to demonstrate 2D lesion distribution of patients with high lesion load and normal g-ratio in the axial plane, g-ratios within WML were averaged in the z-direction; red indicates an abnormally elevated g-ratio and blue is indicative of a normal g-ratio. There is no apparent difference in the distribution of lesion location in the axial plane between low and high g-ratio individuals (Supplementary Figure 4) with a high lesion load.

There was no significant difference in lesion volume between individuals with high and normal WML g-ratios for either the high lesion load group (0.998 vs 1.158, W=185, p=0.72, Wilcoxon Rank Sum Test) or the low lesion load group (0.299 vs 0.180, W=196, p=0.07).

Within the high lesion load group, the anatomical location of lesions did not differ, when assessed visually on 2D “flattened” lesion distribution maps

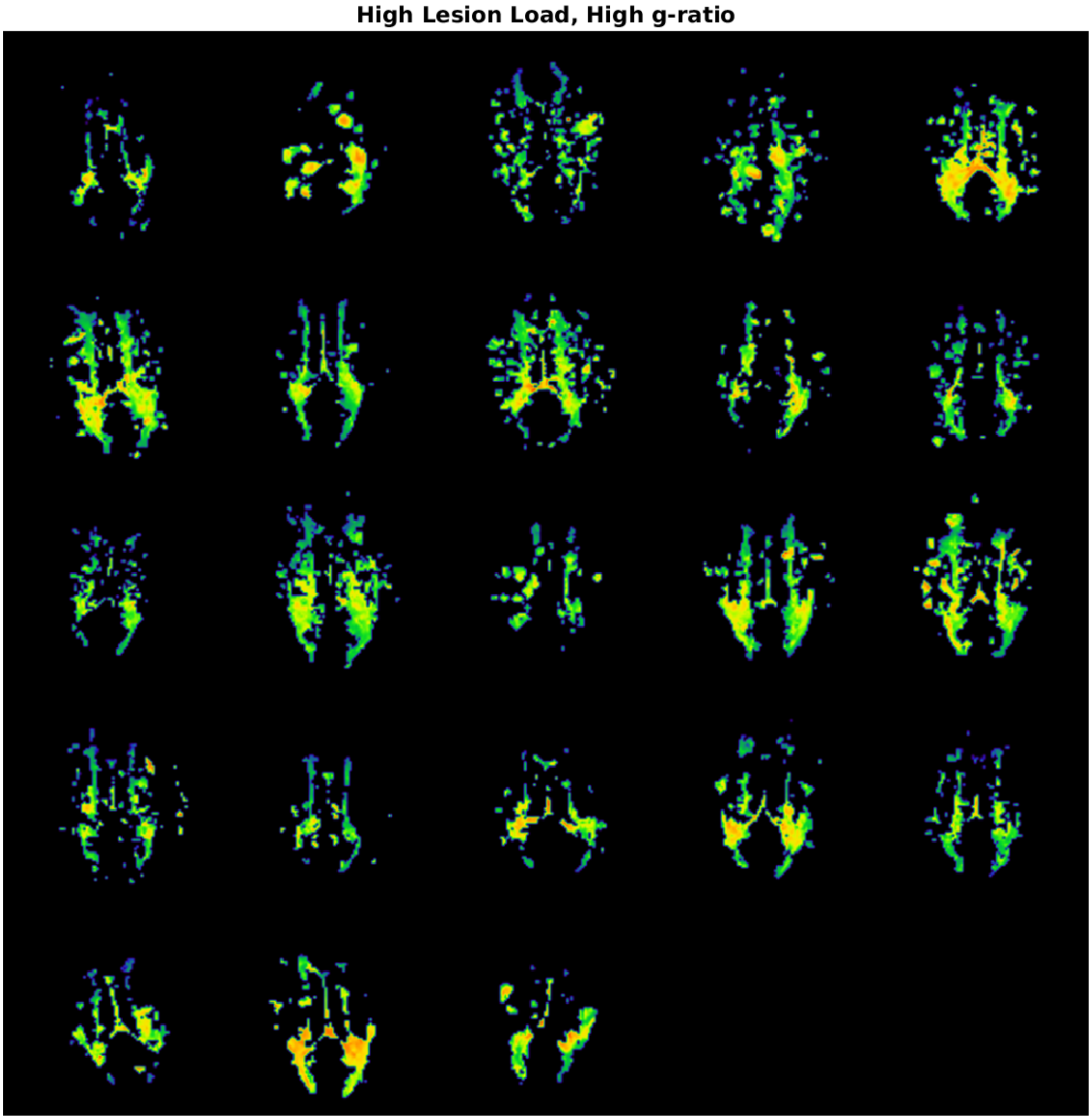

Supplementary Figure 4. Flat map images of individual study participants with high lesion load (Corrected WML volume> 0.5%) and high g-ratio

As in Supplementary Figure 3 distribution of WML g-ratio in patients with high lesion load and abnormal g-ratio in the axial plane is illustrated by averaging WML g-ratio in the z-direction. Again, red indicates an abnormally elevated g-ratio and blue is indicative of a normal g-ratio. There is no apparent difference in the distribution of lesion location in the axial plane between low (Supplementary Figure 3) and high g-ratio individuals with a high lesion load.

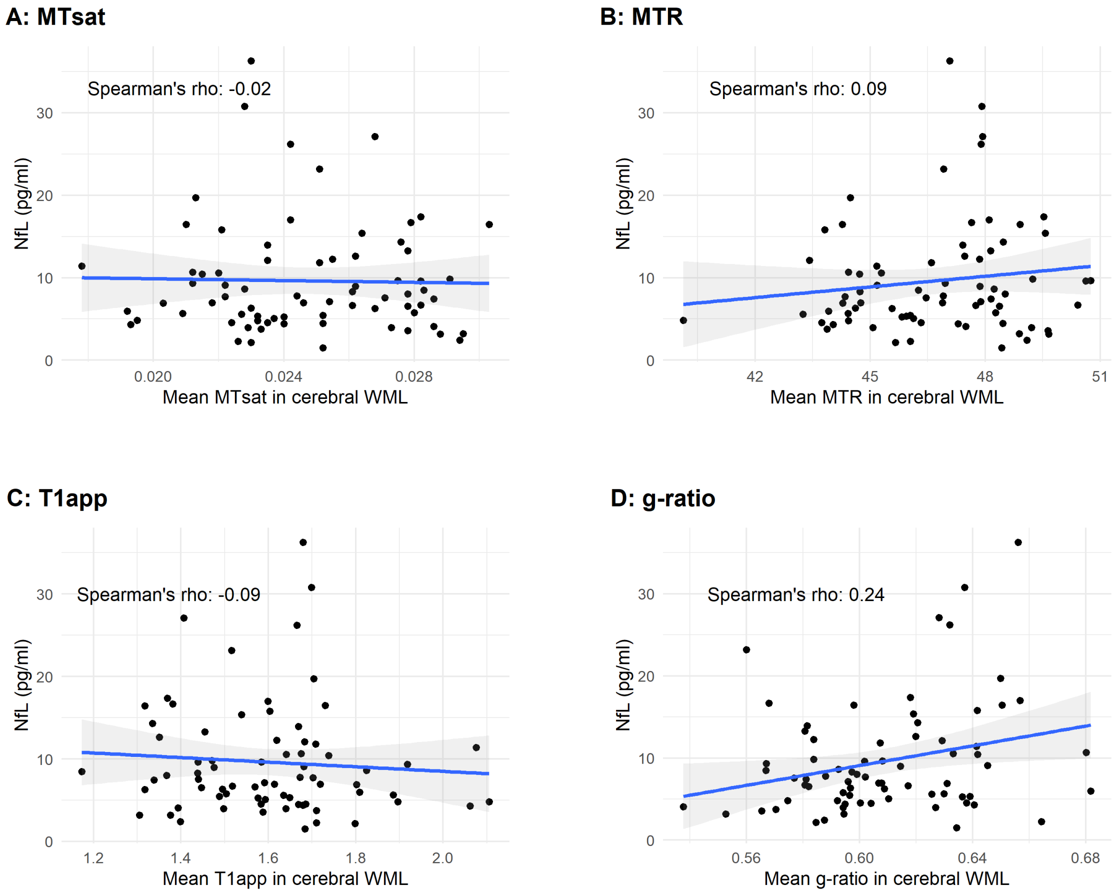

Supplementary Figure 5. Correlation of MTsat, MTR, T1app and g-ratio with serum NFL

In nonparametric tests of correlation (Spearman's rho) g-ratio is significantly associated with NFL (r_s=0.24, p<0.05). This was not observed in MTSat, MTR and T1app analyses

<table>
<tr><th colspan="12">Supplementary Table MRI Acquisition Parameters</th></tr>
<tr><td colspan="12">3.0-T MR system (Prisma; Siemens Healthcare, Erlangen, Germany) with a 32-channel head coil</td></tr>
<tr><th colspan="2">Sequence</th><th>FOV</th><th>Acquisition Matrix</th><th>Slices</th><th>Voxel dimensions</th><th>TE (ms)</th><th>TR (ms)</th><th>α (deg)</th><th>TI (ms)</th><th>In-plane x slice acceleration factor</th><th>Acquisition time (mm:ss)</th></tr>
<tr><td colspan="2">sagittal 3D T1-weighted MPRAGE</td><td>256 x 256</td><td>256 x 256</td><td>176</td><td>1mm isotropic</td><td>2.26</td><td>2500</td><td>7</td><td>1100</td><td>2 x 1</td><td>5:59</td></tr>
<tr><td colspan="2">axial 2D T2 FLAIR</td><td>250 x 250</td><td>250 x 250</td><td>60</td><td>1 x 1 x 3mm</td><td>120</td><td>9500</td><td>150</td><td>2400</td><td>2 x 1</td><td>4:47</td></tr>
<tr><td rowspan="3">sagittal 3D FLASH spoiled gradient echo</td><td>proton density (no saturation pulse)</td><td rowspan="3">224 (sagittal-inferior) x 241 (anterior-posterior)</td><td rowspan="3">160 x 172</td><td rowspan="3">128</td><td rowspan="3">1.4 mm isotropic</td><td rowspan="3">1.54/ 4.55/ 8.49</td><td rowspan="2">30</td><td rowspan="2">5</td><td rowspan="3">-</td><td rowspan="3">2 x 1</td><td>6:14</td></tr>
<tr><td>proton density (with saturation pulse)</td><td>6:14</td></tr>
<tr><td>T1-weighted (no saturation pulse)</td><td>15</td><td>18</td><td>3:08</td></tr>
<tr><td colspan="2"></td><td colspan="10">magnetisation transfer gaussian off-resonance saturation pulse (α: 500°; duration: 9.984 ms; offset: 1.2kHz)</td></tr>
<tr><td colspan="2">axial multiband diffusion-weighted echo-planar imaging</td><td>256 x 256</td><td>128 x 128</td><td>74</td><td>2.0 mm isotropic</td><td>74</td><td>4300</td><td>-</td><td>-</td><td>2 x 2</td><td>11:12</td></tr>
<tr><td colspan="2"></td><td colspan="10">acquired with 151 diffusion directions comprising interleaved b-values of 0 (14), 200 (3), 500 (6), 1000 (64), 2000 (64) s/mm², in addition to 3 x b0 volumes with reversed phase encoding</td></tr>
<tr><td colspan="12">FOV: field of view; SI: sagittal-inferior; AP: anterior-posterior; TE: echo time; TR: repetition time; MPRAGE: Magnetisation Prepared Rapid Gradient Echo; FLASH: fast low-angle shot spoiled gradient echo</td></tr>
</table>

Supplementary Table 1. MRI Acquisition Parameters

	LOW LESION VOLUME			HIGH LESION VOLUME		
	LOW g-ratio		HIGH g-ratio	LOW g-ratio		HIGH g-ratio
**Baseline EDSS**	2		2.5	2		2.5
**1-year EDSS**	2		3	3		3

Supplementary Table 2. Median baseline and 1-year EDSS scores for study individuals, according to lesion volume and g-ratio

There was no correlation between g-ratio and baseline EDSS (Spearman's rho=0.08, p=0.51), and no correlation between NfL and EDSS (Spearman's rho=0.06, p=0.63)

Fisher's Exact Test: Threshold: 0.4%				
	High Lesion Load (n=43)		Low Lesion Load (n=30)	
	Normal g-ratio	High g-ratio	Normal g-ratio	High g-ratio
Normal NfL	15	15	17	9
High NfL	2	11	3	1
p-value	0.045		1	

Fisher's Exact Test: Threshold: 0.5%				
	High Lesion Load (n=38)		Low Lesion Load (n=35)	
	Normal g-ratio	High g-ratio	Normal g-ratio	High g-ratio
Normal NfL	13	12	19	12
High NfL	2	11	3	1
p-value	0.039		1	

Fisher's Exact Test: Threshold: 0.6%				
	High Lesion Load (n=33)		Low Lesion Load (n=40)	
	Normal g-ratio	High g-ratio	Normal g-ratio	High g-ratio
Normal NfL	12	10	20	14
High NfL	1	10	4	2
p-value	0.022		1	

Supplementary Table 3. Sensitivity analysis for lesion volume threshold

Appendix - Members of the Future-MS Consortium

The FutureMS consortium includes clinicians, data processors, or other FutureMS team members that have contributed to acquisition of data, access to patients, or whom have been relied upon for their input.

Location	Affiliations
**Edinburgh**	
Siddharthan Chandran	Anne Rowling Clinic, Centre for Clinical Brain Sciences, Edinburgh Neuroscience, Euan MacDonald Centre for MND Research, University of Edinburgh, NHS Lothian
Peter Connick	Anne Rowling Clinic, Centre for Clinical Brain Sciences, University of Edinburgh, NHS Lothian
David Hunt	Anne Rowling Clinic, Centre for Clinical Brain Sciences, Institute of Genetics and Cancer, University of Edinburgh, NHS Lothian,
Christine Batchelor	Anne Rowling Clinic, Centre for Clinical Brain Sciences, University of Edinburgh
Sara Hathorn	Anne Rowling Clinic, Centre for Clinical Brain Sciences, University of Edinburgh
Denise Cranley	Anne Rowling Clinic, Centre for Clinical Brain Sciences, University of Edinburgh
Mary Monaghan	Anne Rowling Clinic, Centre for Clinical Brain Sciences, University of Edinburgh
Shuna Colville	Anne Rowling Clinic, Centre for Clinical Brain Sciences, University of Edinburgh
Suzanne Quigley	Anne Rowling Clinic, Centre for Clinical Brain Sciences, University of Edinburgh
Kiran Jayprakash	Anne Rowling Clinic, Centre for Clinical Brain Sciences, University of Edinburgh
Elizabeth Elliot	Anne Rowling Clinic, Centre for Clinical Brain Sciences, University of Edinburgh
Patrick Kearns	Anne Rowling Clinic, Centre for Clinical Brain Sciences, University of Edinburgh
Michaela Kleynhans	Anne Rowling Clinic, Centre for Clinical Brain Sciences, University of Edinburgh

Fraser Brown	Anne Rowling Clinic, Centre for Clinical Brain Sciences, University of Edinburgh
Stella Glasmacher	Anne Rowling Clinic, Centre for Clinical Brain Sciences, University of Edinburgh
Lee Murphy	Welcome Trust Genetics Core Laboratory, University of Edinburgh
Alan Maclean	Welcome Trust Genetics Core Laboratory, University of Edinburgh
Katarzyna Hafezi	Welcome Trust Genetics Core Laboratory, University of Edinburgh
Peter Foley	Anne Rowling Clinic, Centre for Clinical Brain Sciences, University of Edinburgh, NHS Lothian
Don Mahad	Anne Rowling Clinic, Centre for Clinical Brain Sciences, University of Edinburgh, NHS Lothian
Belinda Weller	Anne Rowling Clinic , University of Edinburgh, NHS Lothian
Nicola Macleod	Anne Rowling Clinic , University of Edinburgh, NHS Lothian
Emily Harrison	Anne Rowling Clinic , University of Edinburgh, NHS Lothian
Matt Justin	Anne Rowling Clinic , University of Edinburgh, NHS Lothian
Anna Williams	Anne Rowling Clinic, Centre for Clinical Brain Sciences, University of Edinburgh, NHS Lothian
Katy Murray	Anne Rowling Clinic, Centre for Clinical Brain Sciences, University of Edinburgh, NHS Lothian
Dawn Lyle	Anne Rowling Clinic, Centre for Clinical Brain Sciences, University of Edinburgh, NHS Lothian
Judith Newton	Anne Rowling Clinic, Centre for Clinical Brain Sciences, University of Edinburgh, NHS Lothian
Haane Haagenrud	Anne Rowling Clinic, Centre for Clinical Brain Sciences, University of Edinburgh, NHS Lothian
Emily Beswick	Anne Rowling Clinic, Centre for Clinical Brain Sciences, University of Edinburgh, NHS Lothian
Juan Larraz	Anne Rowling Clinic, Centre for Clinical Brain Sciences, University of Edinburgh, NHS Lothian
Michael Wong	Anne Rowling Clinic, Centre for Clinical Brain Sciences, University of Edinburgh, NHS Lothian
Charis Wong	Anne Rowling Clinic, Centre for Clinical Brain Sciences, University of Edinburgh, NHS Lothian

Jessie Chang	Anne Rowling Clinic, Centre for Clinical Brain Sciences, University of Edinburgh, NHS Lothian
Maria Valdez Hernandez	Anne Rowling Clinic, Centre for Clinical Brain Sciences, University of Edinburgh, NHS Lothian
Lucy Kessler	Edinburgh Imaging, NHS Lothian
Dr Rozanna Meijboom	Centre for Clinical Brain Sciences, University of Edinburgh, Edinburgh Imaging
Professor Adam Waldman	Centre for Clinical Brain Sciences, University of Edinburgh, NHS Lothian, Edinburgh Imaging
Agniete Kampaite	Centre for Clinical Brain Sciences, University of Edinburgh, NHS Lothian, Edinburgh Imaging
Julian Ng Kee Kwong	University of Edinburgh
Daisy Mollison	Centre for Clinical Brain Sciences, University of Edinburgh, NHS Lothian, Edinburgh Imaging
Stewart Wiseman	University of Edinburgh, Demetian Research Institute, Centre for Clinical Brain Sciences, Edinburgh Imaging
Mark Bastin	Centre for Clinical Brain Sciences, University of Edinburgh, NHS Lothian, Edinburgh Imaging
Elizabeth York	Centre for Clinical Brain Sciences, University of Edinburgh, NHS Lothian, Edinburgh Imaging
Yingdi Chen	Anne Rowling Clinic, Centre for Clinical Brain Sciences, University of Edinburgh, NHS Lothian
Baljean Dhillon	Centre for Clinical Brain Sciences<br>Division of Health Sciences<br>NHS Lothian
Michael Thrippleton	Centre for Clinical Brain Sciences, University of Edinburgh, NHS Lothian, Edinburgh Imaging
Aidan Hutchinson	Anne Rowling Clinic ,Centre for Clinical Brain Sciences, University of Edinburgh
David Perry	Centre for Clinical Brain Sciences, University of Edinburgh
Angus Grossart	Anne Rowling Clinic ,Centre for Clinical Brain Sciences, University of Edinburgh
Amy Stenson	Anne Rowling Clinic , University of Edinburgh, NHS Lothian
Christine Weaver	Anne Rowling Clinic, Centre for Clinical Brain Sciences, University of Edinburgh, NHS Lothian

Rachel Dakin	Anne Rowling Clinic, Centre for Clinical Brain Sciences, University of Edinburgh, NHS Lothian
**Glasgow**	
Stewart Webb	Institute of Neurological Sciences, Glasgow, NHS GGC
Niall MacDougall	Institute of Neurological Sciences, Glasgow, NHS GGC, University of Glasgow
Kathryn Love	Institute of Neurological Sciences, Glasgow, NHS GGC
Sarah Jane Martin	Institute of Neurological Sciences, Glasgow, NHS GGC
Lynn McMahon	Precision Medicine Scotland Innovation Centre, University of Glasgow
**Dundee**	
Jonathan O'Riordan	University of Dundee, NHS Tayside
Lesley Macfarlane	NHS Tayside
Gwen Kennedy	NHS Tayside
Tracey Hopkins	NHS Tayside
**Aberdeen**	
Margaret Ann MacLeod	NHS Grampian
James MacDonald	NHS Grampian
Beverly Maclennan	NHS Grampian
**Inverness**	
Javier Carod Artal	NHS Highland
Fiona Barret	NHS Highland
James Finlayson	NHS Highland
Adam Scotson	NHS Highland
Ian Megson	NHS Highland
**UCSF**	
Sergio Baranzini	University of California San Francisco
Adil Harroud	University of California San Francisco
Amit Akula	University of California San Francisco

References

1. Helms G, Dathe H, Kallenberg K, Dechent P. High-resolution maps of magnetization transfer with inherent correction for RF inhomogeneity and T1 relaxation obtained from 3D FLASH MRI. Magn Reson Med 2008;60:1396-407.
